# Coping with age-related hearing loss: patient-caregiver dyad effects on quality of life

**DOI:** 10.1186/s12955-019-1161-6

**Published:** 2019-05-22

**Authors:** Sébastien Lazzarotto, Florence Martin, Anne Saint-Laurent, Zeinab Hamidou, Valérie Aghababian, Pascal Auquier, Karine Baumstarck

**Affiliations:** 1 EA 3279 CEReSS - Health Service Research and Quality of Life Center, Aix-Marseille Univ, Marseille, France; 2 Centre de Prévention du Bien Vieillir PACA, Marseille, France; 3 Centre de Prévention Bien Vieillir de Toulouse, Marseille, France; 4 Social Action Departement, GIE AGIRC ARRCO, Marseille, France; 5 National Clinical Research Quality of Life in Oncology Platform, Marseille, France

**Keywords:** Age-related hearing loss, Caregivers, Dyads, Interactions, Quality of life, Coping, Emotional status

## Abstract

**Background and aims:**

Patients with age -related hearing loss (ARHL) and their natural caregivers have to confront a disability that produces progressive lifestyle changes. There is an interest in studying the ability of patients and their caregivers to cope with the difficulties that affect quality of life (QoL). In a sample of patient-caregiver dyads in the specific context of ARHL, we examine whether the QoL of patients and caregivers is influenced by the coping processes they use from a specific actor–partner interdependence model (APIM).

**Methods:**

This cross-sectional study involved dyads with patients having a diagnosis of ARHL. The self-reported data included QoL (WHOQoL-BREF) and coping strategies (BriefCope). The APIM was used to test the dyadic effects of coping strategies on QoL.

**Results:**

A total of 448 dyads were included; the patients and caregivers were love partners for 59% of the dyads. Coping strategies, such as social support, avoidance, problem solving, and positive thinking, exhibited evidence of actor effects (degree to which the individual’s coping strategies are associated with their own QoL). Effects on the partner (degree to which the individual’s coping strategies are associated with the QoL of the other member of the dyad) were found, i.e., when the patients mobilized their coping strategy based on social support and problem-solving, their caregivers reported higher environmental QoL.

**Conclusion:**

This study emphasizes that the QoL for patients and their caregivers was directly related to the coping strategies they used. This finding suggests that targeted interventions should be offered to help patients and their relatives to implement more effective coping strategies.

**Electronic supplementary material:**

The online version of this article (10.1186/s12955-019-1161-6) contains supplementary material, which is available to authorized users.

## Background

Hearing impairment is one of the most common disabilities in humans, affecting more than 250 million people worldwide [1, 2]. In developed countries, aging is the most common cause of hearing impairment and is referred to as age-related hearing loss (ARHL) or presbycusis. The consequences of ARHL in daily life include difficulty of interpreting speech sounds, which often results in a reduced ability to communicate, impaired physical and social functions, and an altered quality of life (QoL) [3–7]. Some authors have also reported that ARHL affects relatives’ functioning in everyday life, specifically the main family caregiver [8–10].

The impact on self-reported QoL may differ according to the individuals’ sociodemographic and clinical characteristics and other psycho-behavioral factors. In particular, the personal ability to cope, defined as the cognitive and behavioral efforts that the individuals implement to solve problems and reduce the stress that may result [11–13], has previously been shown as having a direct impact on the QoL of the individuals. More interesting, the nature of individuals’ coping strategies may have a direct impact on the QoL [14] of their natural caregivers as shown in various contexts, such as cancer [15, 16] or severe mental disease [17]. These different studies showed that positive thinking or problem solving seem to be preferentially associated with better QoL, and the use of strategies based on avoidance or social support appear to be a risk factor for lower QoL [15–18].

In the specific context of ARHL, we previously emphasized [19] that quality of life of individuals and their natural caregivers was related to the coping strategies that they use. However, the previous study was performed on a small sample size (*N* = 44) and did not use specific dyadic analyses that integrate a conceptual view of interdependence in two-person relationships. Therefore, to consider the reciprocal influences and congruence between the patients and their caregivers, studies should be conducted at the ‘dyadic level’. Thus, among a large sample of patient-caregiver dyads, we examined whether the QoL of patients and caregivers is influenced by the coping strategies implemented by either themselves or their relatives, using the actor–partner interdependence model (APIM) [20]. This approach, as an appropriate method to assess the dyadic effects, is based on the hypothesis that the scores within the same dyad are not independent but instead are more similar than the scores of two individuals who are not in the same dyad.

## Methods

### Design and settings

We conducted a cross-sectional study with a descriptive, correlational design. Patient-caregiver dyads were recruited in the 17 French preventive health centers for the beneficiaries of complementary pension schemes for employees of the private sector (Association Générale des Institutions de Retraite Complémentaire des cadres and l’Association pour le Régime de Retraite Complémentaire des salaries, AGIRC-ARRCO, http://www.agirc-arrco.fr). These centers perform 25,000 annual health checkups for beneficiaries and/or partners of these beneficiaries, including medical, psychosocial, and cognitive prevention. The visits last a half day. Regulatory monitoring was performed in accordance with the French law requiring the approval of the French ethics committee (Comité d’éthique, Aix Marseille University, February 4th 2015, Number 2014 − 02 − 04 − 04). All records and subjects’ identities remained confidential in accordance with French regulations: the French National Committee of Informatics and Liberties (Commission nationale de l’informatique et des libertés, 20/03/2014, reference number DR-2014 − 097). Consent was obtained from each participant.

### Sample selection

The samples included patient-caregiver dyads. The selection criteria of the patients were as follows: aged above 65 years; having a bilateral sensorineural ARHL and having a degree of hearing loss from mild (≥21 dB, dB HL) to moderately severe (< 70 dB HL) according to the Clark’s classification [21] and to the International Bureau for Audiophonology Classification (https://www.biap.org/fr/archives/65-ct-2-classification-des-surdites) (hearing loss was defined from the mean hearing loss of the 4 following frequencies: 500, 1000, 2000, and 4000 Hz); being beneficiaries of complementary pension schemes for employees of the private sector (AGIRC-ARRCO); able to speak/read French; and agreeing to participate. The selection criteria of the caregivers were as follows: aged above 18 years; designated by the patient as the most involved person in his/her life; able to speak/read French; and agreeing to participate. In this context, ‘caregiver’ is close to ‘significant other’ as defined by the World Health Organization’s International Classification of Functioning [22]. Written consent forms to participate were collected from every patient and caregiver.

### Data collection

Inclusions were performed during a 4-month period. For the patient, the clinical data were gathered using the medical records and the examination by a geriatric doctor of the center. The nature of the relationship between the patient and the caregiver was recorded (love partner, child, or other). The age, gender, educational level, marital status, professional status, and health status (chronic disease, cancer, and stroke) were recorded for both the patient and his/her caregiver using self-report questions. The hearing severity was recorded for the patients (two levels of hearing severity: < or ≥ 35 dB HL).

Quality of life and coping strategies were collected by means of self-reported questionnaires that were completed by the patients and caregivers. On average, it took participants 25 min to complete all study assessments.

- Quality of life was assessed using the French version of the World Health Organization Quality of Life (WHOQoL-BREF) questionnaire, which is a generic, 26-item questionnaire used worldwide to assess the effects of physical health, psychological health, social health, and environmental factors on QoL [23, 24]. All scores range from 0 (worst QoL) to 100 (best QoL).
- Coping strategies were assessed using the Brief Coping Orientation to Problems Experienced Scale (BriefCope) [25, 26]. This questionnaire includes 28 items that explore the following 14 strategies: self-distraction, active coping, denial, substance use, use of emotional support, use of instrumental support, behavioral disengagement, venting, positive reframing, planning, humor, acceptance, religion, and self-blame. A four-factor structure has shown satisfactory properties [27]: social support, problem solving, avoidance, and positive thinking (Additional file 1: Dimensions and items of the French four-factor structure of the Brief COPE inventory). Scores range from 0 to 100. Higher scores in the 4 dimensions reflect a high tendency to implement the corresponding coping strategies.

### Statistical analyses

After descriptive analyses of the characteristics of patients and caregivers, QoL scores were computed using the algorithms provided by the respective developers of the tools. The WHOQoL scores of patients and caregivers were compared to those obtained from French age- and sex-matched controls from a normal sample of 16,392 subjects [24]. To assess the relationships between the QoL scores and coping processes for patients and caregivers, two analyses were performed: i) Paired t-tests were used to compare various scores between patients and caregivers (multiple comparison corrections, false discovery rate); and ii) an analysis based on the actor–partner interdependence model (APIM) to assess the dyadic effects of coping strategies on QoL (WHOQoL scores). The APIM was assessed using structural equation modeling [20]. This model is based on the fact that the scores within the same dyad are not independent but instead are more similar than the scores of two individuals who are not in the same dyad. The APIM is useful to determine how parameters (QoL and coping strategies) among each participant (namely, patients and caregivers) are influenced by not only internal factors but also factors related to the other member of the dyad. Structural equation modeling simultaneously examines both paths in the APIM: two actor effects (i.e., each person’s QoL regressed on his or her own coping strategies) and two partner effects (i.e., each person’s QoL regressed on the other person’s coping strategies). Using the same procedure, the APIM analyses were performed on the subsample of dyads with a love partner relationship.

## Results

### Sample

Between November 2015 and February 2016, 497 patients were identified as eligible to be included in the cohort. Of the 497 patients, 483 were able to nominate a caregiver who agreed to participate. The final sample was composed of 448 patients and 448 caregivers. The relationships between the patients and the caregivers were as follows: 262 were love partners and 51 were children/parents (41 dyads had other kinds of relationships, such as siblings, friends, or parent/stepchild, and the information was lacking for 94 dyads). None of the individuals had a diagnosis of dementia at the time of the evaluation. The main characteristics are presented in Table 1.

**Table 1:** Characteristics of the 448 patient-caregiver dyads

		Patients	Caregivers
		N (%)	N (%)
Age, years	M ± SD	71.4 ± 6.8	67.9 ± 11.4
	m [IQR]	70 [66–77]	69 [63–76]
Gender	Woman	204 (46)	276 (62)
	Man	242 (54)	170 (38)
Education level	< 12 years	200 (45)	196 (44)
	≥ 12 years	240 (55)	247 (56)
Marital status	Single	67 (15)	48 (11)
	Couple	379 (85)	399 (89)
Professional status	Worker	67 (15)	84 (21)
	Not worker	372 (85)	360 (79)
Chronic disease^a^	No	223 (51)	202 (46)
	Yes	214 (49)	236 (54)
Cancer	No	374 (84)	428 (97)
	Yes	69 (16)	72 (12)
Stroke	No	425 (96)	428 (97)
	Yes	18 (4)	14 (3)
Hearing severity	< 35 dB HL	283 (70)	
	≥35 dB HL	119 (30)	

*N (%)* effective (percents), *M ± SD* mean ± standard deviation, *m [IQR]* median [interquartile range]

^a^excluding cancer

### Quality of life and coping strategies of the patients and caregivers

**Fig. 1:**
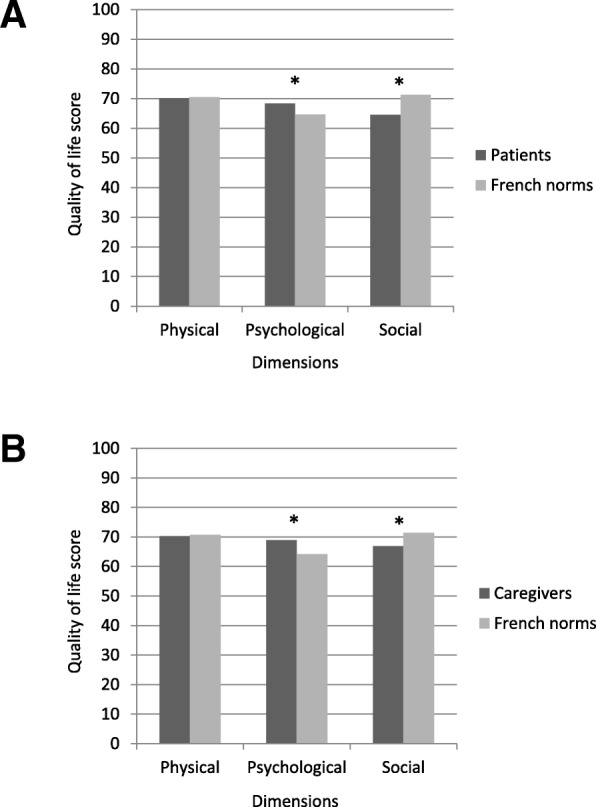
Comparisons of WHOQoL scores between the participants and French age-sex-matched norms ^#^. Higher the scores, higher the QoL level. ^**#**^ Baumann C, Erpelding ML, Regat S, Collin JF, Briancon S: The WHOQOL-BREF questionnaire: French adult population norms for the physical health, psychological health and social relationship dimensions. Rev. Epidemiol Sante Publique 2010, 58(1):33–39. *p < 0.05 (Wilcoxon paired test). **a** Patients, **b** Caregivers

The QoL scores of patients and caregivers are provided in Fig. 1 (a and b, respectively). Of the 3 dimensions of the WHOQoL for which French norms are available, the scores of the physical dimension did not differ from the age- and sex-matched controls. For both the patients and the caregivers, the scores of the psychological dimension were statistically higher than norms, and the scores of the social dimension were statistically lower than norms.

Patients and caregivers used the four types of coping strategies at the same level. The strategies that were based on social support and avoidance were lesser used (from 23 to 33) than strategies based on problem solving and positive thinking (from 44 to 58) among patients and caregivers.

### Relationships between coping strategies and quality of life within the dyads

- Assessment of relationships between patients’ and caregivers’ scores

The correlations between patients’ and caregivers’ scores are given in Table 2. When patients used coping strategies such as social support, problem solving and positive thinking, they reported significantly higher QoL scores in almost all dimensions, and when they used strategies based on avoidance, they reported significantly lower QoL scores. For the caregivers, using problem-solving and positive-thinking strategies was linked to higher QoL levels, but being avoidant was linked to lower physical and psychological QoL. We found some interrelations between coping strategies of patients and their caregivers’ QoL. The caregiver reported significantly better QoL in all dimensions when the patient used positive-thinking strategies, better environmental QoL when the patient used strategies based on social support and problem-solving, and better psychological QoL when the patient implemented problem-solving. We found some interrelations between coping strategies used by the caregivers and their patients’ QoL. The patients reported higher QoL when the caregivers used social support and problem-solving strategies in some dimensions.

- Actor–partner interdependence model to assess the dyadic effects

**Table 2:** Paired t test correlations between QoL and coping strategies

		Patients’ QoL (WHOQoL scores)				Caregivers’ QoL (WHOQoL scores)			
		Physical	Psychol.	Social	Environ.	Physical	Psychol.	Social	Environ.
Patients’ coping (BriefCope)	Social support	−0,035	0,000	**0,138** ^**^	**0,105** ^*^	0,040	0,059	0,002	**0,149** ^**^
	Problem-solving	**0,263** ^**^	**0,343** ^**^	**0,182** ^**^	**0,293** ^**^	0,076	**0,107** ^*^	0,071	**0,121** ^*^
	Avoidance	**−0,227** ^**^	**−0,342** ^**^	**−0,221** ^**^	**− 0,166** ^**^	0,012	− 0,043	− 0,058	−0,076
	Positive-thinking	**0,271** ^**^	**0,384** ^**^	**0,296** ^**^	**0,327** ^**^	**0,112** ^*^	**0,114** ^*^	**0,128** ^**^	**0,130** ^**^
Caregivers’coping (BriefCope)	Social support	**0,100** ^*^	**0,113** ^*^	−0,012	**0,098** ^*^	0,021	0,012	0,060	0,087
	Problem-solving	0,021	0,040	**0,098** ^*^	0,078	**0,304** ^**^	**0,386** ^**^	**0,223** ^**^	**0,290** ^**^
	Avoidance	−0,065	−0,021	-0,056	-0,059	**-0,134** ^**^	**-0,239** ^**^	-0,079	-0,093
	Positive-thinking	-0,039	-0,015	0,004	0,030	**0,287** ^**^	**0,458** ^**^	**0,254** ^**^	**0,300** ^**^

*BriefCope* higher the scores, higher use of the strategy, *WHOQoL* higher the scores, higher the QoL, *Bold values* significant correlation; *p < 0.05, **p < 0.01

**Fig. 2:**
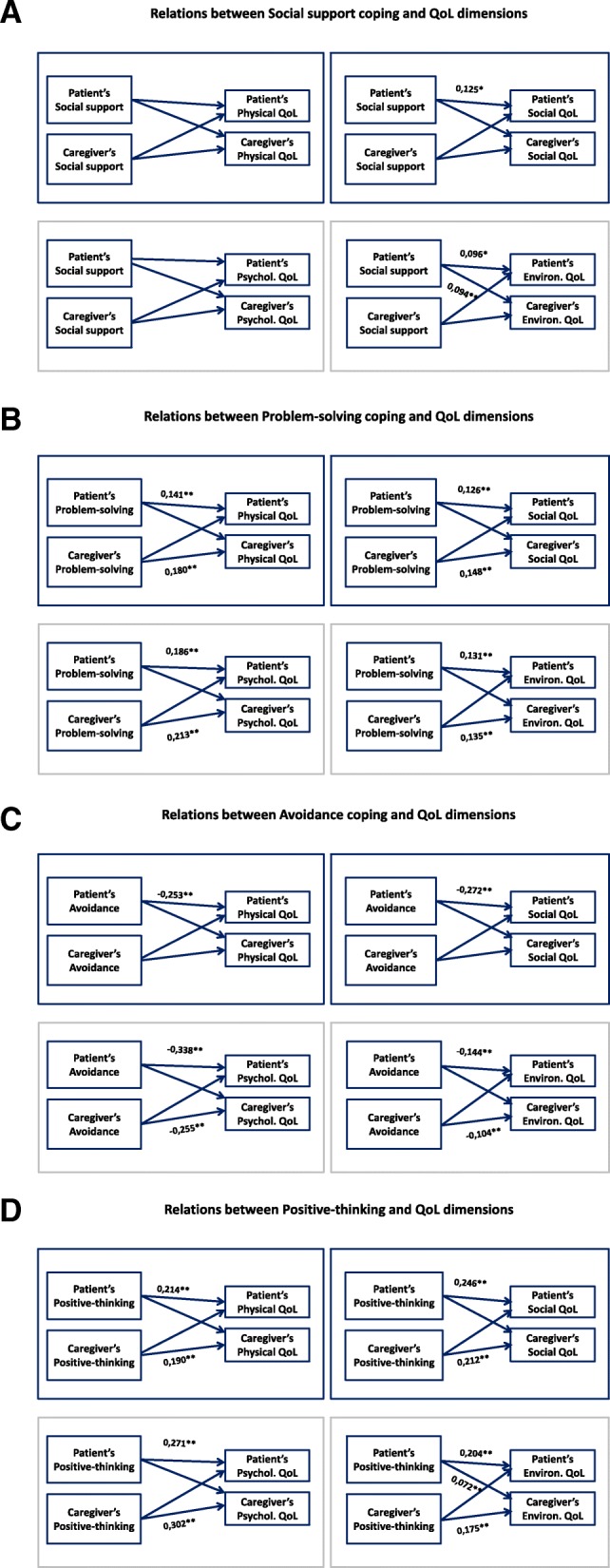
Illustration of relations between coping and QoL using the actor–partner interdependence model. Numbers are standardized β coefficients: *p < .05; **p < .01

Figure 2 presents the results of the APIM analysis conducted to establish any associations between coping strategies (4 dimensions of BriefCope) and QoL (4 dimensions of WHOQoL), considering the dyadic effects, based on the fact that the scores within the same dyad are not independent but instead are more similar than the scores of two individuals who are not in the same dyad. The following actors’ effects were found: the use of problem-solving (Fig. 2b) and positive-thinking (Fig. 2d) strategies was systematically associated with an increase in their own QoL scores, for both patients and caregivers; the use of avoidance strategies (Fig. 2c) by patients and caregivers was associated with a decrease of their own QoL scores; and the patient’s coping strategy to seek social support (Fig. 2a) was associated with a decrease in their own QoL social and environmental scores. Two partner effects were observed: the use of social support (Fig. 2a) and the use of problem-solving (Fig. 2d) by the patient were linked to a higher environmental QoL of the caregiver. Similar findings were found on the subsample of dyads with a love partner relationship (data not shown).

## Discussion

This study is one of the few that have used a large sample of ARHL patient-caregiver dyads to describe the relationship between individuals’ coping strategies and their self-reported QoL. We used specific dyadic analyses based on the APIM, which was specifically developed to evaluate two-person relationships that integrate a conceptual view of independence in such relationships. The results lead to the reassessment of previous results from patient-only evaluations.

The first finding of this study indicates that ARHL patients and their caregivers similarly presented a significant negative impact on the social dimension of the QoL. Indeed, the hearing disability may lead to communicative disability, relative isolation, and reduced social activity [3]. This progressive social deprivation that the ARHL individual imposes on him/herself may entrain a broader social deprivation of one’s closest relatives [28, 29]. These findings indicate the importance of early identification of hearing loss and offers of rehabilitative support [30]. Less expected, both for patients and caregivers, we found better QoL scores for the physical and psychological dimensions in comparison with age- and sex-matched controls. This result was in opposition with most studies that have reported negative psychological consequences of the hearing disability as feelings of being excluded, mood disorders, and depression [31]. This assumption was recently moderated through studies indicating that the relationships between hearing impairment and depressive symptoms differ according to the availability of social resources [32]. We may hypothesize that the people involved in this study, who voluntarily underwent a health checkup, indicating that they were concerned about prevention and health promotion, have the highest level of resources. In contrast, many non-participants in this study, including those living in isolation, those in denial about their impairment, and those who are reluctant to seek help from the health care system, are likely to have lower levels of support resources than our study participants.

The second interesting result of this study is that patients and their caregivers were found to implement similar coping strategies, using strategies based on problem solving and positive thinking more than the strategies based on seeking social support or avoidance. While the coping strategies employed depend on the cognitive, behavioral and social resources that the patients and caregivers are able to mobilize, this result suggests that people who know each other very well and who are faced with the same difficult event tend to cope with it similarly. This phenomenon was already described in the context of ARHL [19], but has also been described in other contexts, such as cancer [15, 16] and mental illnesses [17].

Interestingly, we observed that the nature of the individuals’ coping strategies may have a direct impact on their QoL and their relatives’ QoL. First, individuals (the ARHL patients and the caregivers) who used problem-solving or positive-thinking strategies systematically reported higher QoL. These strategies are known to be better methods to address stressful events. In the same way, individuals who mobilized avoidant coping strategies, known as incorrect responses to stressful life events [33–35], had lower QoL levels. A systematic assessment of patient and caregiver coping styles may identify individuals who do not adopt healthy coping strategies. Targeted psychological interventions based on psychoeducation and cognitive behavioral therapy may be offered to them [36, 37] to maintain a stable QoL level [38]. Interventions based on optimism or positive thinking have revealed satisfactory well-being predictors on not only the individuals themselves [39–41] but also their relatives [42].

Lastly, the APIM approach described partner effects related to the use of social support coping strategies. As previously reported [43], the use of social support by the caregiver or the patient may positively impact the QoL of the second member of the dyad. Some hypotheses may be proposed: i) individuals may feel relieved when they realize that the second member of the dyad implemented social support and sought external help; and ii) individuals may be more able to pay attention, to understand and to support their relatives when they themselves are helped by an external process. Social support may be a relevant factor on which to focus in auditory rehabilitation programs [44]. The involvement of significant others in counseling seems to improve the everyday life for both older adults with age-related hearing loss and their significant others [45]. The approaches based on family-centered care are hopeful strategies for improving QoL [46].

### Strengths and limitations

The generalization of our findings should be done cautiously. It is known that some individuals deny the existence of hearing impairment and do not wish to be managed [47]. Replication of our findings in these groups is required.

The study employed an observational and cross-sectional design, which did not allow for causality inferences to be made between coping strategies and QoL. Thus, it remains unknown whether an individual’s coping strategies actually influence QoL and that of his/her caregivers over time. Specific APIM-based dyadic analyses, which integrate a conceptual view of interdependence in two-person relationships [48–50], are best undertaken within longitudinal designs, as they allow for direct causality inferences to be made.

Some parameters were not analyzed, such as the use of hearing aids or the hearing status of the caregiver. Yet, these parameters may be factors influencing some of the actor-partner effects [51]. Future studies should specifically explore these cases.

## Conclusion

This study emphasizes that the quality of life for patients and their natural caregivers is directly related to the coping strategies that they use. This finding suggests that targeted interventions should be offered to help patients and their caregivers who experience life difficulties to implement more efficient coping strategies.

## Additional file

Additional file 1: Dimensions and items of the French four-factor structure of the Brief COPE inventory. (DOCX 30 kb)

## Abbreviations

AGIRC-ARRCO: Association Générale des Institutions de Retraite Complémentaire des cadres and l’Association pour le Régime de Retraite COmplémentaire des salaries
APIM: Actor–Partner Interdependence Model
ARHL: Age-Related Hearing Loss
QoL: Quality of Life

## References

[1] Global burden of hearing loss in the year 2000. In: Global burden of disease (online). https://www.who.int/healthinfo/statistics/bod_hearingloss.pdf.

[2] DREES (2014). Étude quantitative sur le handicap auditif à partir de l’enquête « Handicap-Santé ». Haeusler, De Laval Th, Millot L. Série études et recherches N°.

[3] Dalton DS, Cruickshanks KJ, Klein BE, Klein R, Wiley TL, Nondahl DM (2003). The impact of hearing loss on quality of life in older adults. Gerontologist.

[4] Mulrow CD, Aguilar C, Endicott JE, Velez R, Tuley MR, Charlip WS, Hill JA (1990). Association between hearing impairment and the quality of life of elderly individuals. J Am Geriatr Soc.

[5] Campbell VA, Crews JE, Moriarty DG, Zack MM, Blackman DK. Surveillance for sensory impairment, activity limitation, and health-related quality of life among older adults--United States, 1993-1997. MMWR CDC Surveill Summ. 1999, 48:131–56. 10634273

[6] Granberg S, Pronk M, Swanepoel de W, Kramer SE, Hagsten H, Hjaldahl J, Moller C, Danermark B (2014). The ICF core sets for hearing loss project: functioning and disability from the patient perspective. Int J Audiol.

[7] Punch JL, Hitt R, Smith SW (2019). Hearing loss and quality of life. J Commun Disord.

[8] Hétu R, Jones L, Getty L (1993). The impact of acquired hearing impairment on intimate relationships: implications for rehabilitation. Audiology.

[9] Piercy SK, Piercy FP (2002). Couple dynamics and attributions when one partner has an acquired hearing loss: implications for couple therapy. J Marital Fam Ther.

[10] Scarinci N, Worrall L, Hickson L (2009). The effect of hearing impairment in older people on the spouse: development and psychometric testing of the significant other scale for hearing disability (SOS-HEAR). Int J Audiol.

[11] Lazarus M, Folkman S: Stress, appraisal and coping. Springer edn. New York; 1984.

[12] Folkman S, Moskowitz JT (2000). Positive affect and the other side of coping. Am Psychol.

[13] Holahan CJ, Moos RH (1987). Personal and contextual determinants of coping strategies. J Pers Soc Psychol.

[14] Baider L, Kaplan De-Nour A (1997). Psychological distress and intrusive thoughts in cancer patients. J Nerv Ment Dis.

[15] Baumstarck K, Leroy T, Hamidou Z, Tabouret E, Farina P, Barrie M, Campello C, Petrirena G, Chinot O, Auquier P (2016). Coping with a newly diagnosed high-grade glioma: patient-caregiver dyad effects on quality of life. J Neuro-Oncol.

[16] Hamidou Z, Auquier P, Leroy T, Barlesi F, Salas S, Chinot O, Baumstarck K (2018). Dyadic effects of coping strategies, time perspectives, and personality on the quality of life of cancer patients and their caregivers. Psychooncology.

[17] Boyer L, Baumstarck K, Alessandrini M, Hamidou Z, Testart J, Serres M, Arquilliere P, Auquier P, Leroy T, Zendjidjian X (2017). Emotional intelligence and coping strategies as determinants of quality of life in depressed patient-caregiver dyads: an actor-partner interdependence analysis. Compr Psychiatry.

[18] Baumstarck K, Chinot O, Tabouret E, Farina P, Barrie M, Campello C, Petrirena G, Hamidou Z, Auquier P (2018). Coping strategies and quality of life: a longitudinal study of high-grade glioma patient-caregiver dyads. Health Qual Life Outcomes.

[19] Lazzarotto S, Baumstarck K, Loundou A, Hamidou Z, Aghababian V, Leroy T, Auquier P (2016). Age-related hearing loss in individuals and their caregivers: effects of coping on the quality of life among the dyads. Patient Prefer Adherence.

[20] Cook WL, Kenny DA (2005). The actor-partner interdependence model: a model of bidirectional effects in developmental studies. Int J Behav Dev.

[21] Clark JG (1981). Uses and abuses of hearing loss classification. Asha.

[22] Scarinci Nerina, Worrall Linda, Hickson Louise (2009). The ICF and third-party disability: Its application to spouses of older people with hearing impairment. Disability and Rehabilitation.

[23] WHOQOL (1998). Group. Development of the World Health Organization WHOQOL-BREF quality of life assessment. The WHOQOL group. Psychol Med.

[24] Baumann C, Erpelding ML, Regat S, Collin JF, Briancon S (2010). The WHOQOL-BREF questionnaire: French adult population norms for the physical health, psychological health and social relationship dimensions. Rev Epidemiol Sante Publique.

[25] Carver CS (1997). You want to measure coping but your protocol's too long: consider the brief COPE. Int J Behav Med.

[26] Muller L, Spitz E (2003). Multidimensional assessment of coping: validation of the brief COPE among French population. Encephale.

[27] Baumstarck K, Alessandrini M, Hamidou Z, Auquier P, Leroy T, Boyer L (2017). Assessment of coping: a new french four-factor structure of the brief COPE inventory. Health Qual Life Outcomes.

[28] Preminger JE, Meeks S (2010). The influence of mood on the perception of hearing-loss related quality of life in people with hearing loss and their significant others. Int J Audiol.

[29] Preminger JE, Meeks S (2012). The hearing impairment impact-significant other profile (HII-SOP): a tool to measure hearing loss-related quality of life in spouses of people with hearing loss. J Am Acad Audiol.

[30] Arlinger S (2003). Negative consequences of uncorrected hearing loss--a review. Int J Audiol.

[31] Vesterager V, Salomon G, Jagd M (1988). Age-related hearing difficulties. II. Psychological and sociological consequences of hearing problems--a controlled study. Audiology.

[32] West JS (2017). Hearing impairment, social support, and depressive symptoms among U.S. adults: a test of the stress process paradigm. Soc Sci Med.

[33] Brunault P, Champagne AL, Huguet G, Suzanne I, Senon JL, Body G, Rusch E, Magnin G, Voyer M, Reveillere C, Camus V (2016). Major depressive disorder, personality disorders, and coping strategies are independent risk factors for lower quality of life in non-metastatic breast cancer patients. Psychooncology.

[34] Mohamadi A, Davoodi-Makinejad M, Azimi A, Nafissi S (2016). Personality characteristics in MS patients: the role of avoidant personality. Clin Neurol Neurosurg.

[35] D'Onofrio G, Simeoni M, Rizza P, Caroleo M, Capria M, Mazzitello G, Sacco T, Mazzuca E, Panzino MT, Cerantonio A (2017). Quality of life, clinical outcome, personality and coping in chronic hemodialysis patients. Ren Fail.

[36] Vanderwerker LC, Laff RE, Kadan-Lottick NS, McColl S, Prigerson HG (2005). Psychiatric disorders and mental health service use among caregivers of advanced cancer patients. J Clin Oncol.

[37] Tiemensma J, Kaptein AA, Pereira AM, Smit JW, Romijn JA, Biermasz NR (2011). Coping strategies in patients after treatment for functioning or nonfunctioning pituitary adenomas. J Clin Endocrinol Metab.

[38] Boele FW, Hoeben W, Hilverda K, Lenting J, Calis AL, Sizoo EM, Collette EH, Heimans JJ, Taphoorn MJ, Reijneveld JC, Klein M (2013). Enhancing quality of life and mastery of informal caregivers of high-grade glioma patients: a randomized controlled trial. J Neuro-Oncol.

[39] Leclaire K, Cecil A, LaRussa A, Stuart F, Hemond CC, Healy BC, Chitnis T, Weiner H, Huffman J, Glanz BI (2018). Short report: a pilot study of a group positive psychology intervention for patients with multiple sclerosis. Int J MS Care.

[40] Calandri E, Graziano F, Borghi M, Bonino S (2017). Improving the quality of life and psychological well-being of recently diagnosed multiple sclerosis patients: preliminary evaluation of a group-based cognitive behavioral intervention. Disabil Rehabil.

[41] Roets-Merken LM, Draskovic I, Zuidema SU, van Erp WS, Graff MJ, Kempen GI, Vernooij-Dassen MJ (2015). Effectiveness of rehabilitation interventions in improving emotional and functional status in hearing or visually impaired older adults: a systematic review with meta-analyses. Clin Rehabil.

[42] Ruisoto P, Contador I, Fernandez-Calvo B, Palenzuela D, Ramos F. Exploring the association between optimism and quality of life among informal caregivers of persons with dementia. Int Psychogeriatr. 2018:1–7. 10.1017/S104161021800090X 30017002

[43] Dean G, Orford A, Staines R, McGee A, Smith KJ (2017). Psychosocial well-being and health-related quality of life in a UK population with usher syndrome. BMJ Open.

[44] Singh G, Lau ST, Pichora-Fuller MK (2015). Social support predicts hearing aid satisfaction. Ear Hear.

[45] Moser S, Luxenberger W, Freidl W (2017). The influence of social support and coping on quality of life among elderly with age-related hearing loss. Am J Audiol.

[46] Ekberg K, Meyer C, Scarinci N, Grenness C, Hickson L (2015). Family member involvement in audiology appointments with older people with hearing impairment. Int J Audiol.

[47] Rawool VW, Keihl JM (2008). Perception of hearing status, communication, and hearing aids among socially active older individuals. J Otolaryngol Head Neck Surg.

[48] Kashy DA, Kenny DA (1999). The analysis of data from dyads and groups. Handbook of research methods in social psychology.

[49] Campbell LJ, Kashy DA (2002). Estimating actor, partner, and interaction effects for dyadic data using PROC MIXED and HLM5: a brief guided tour. Pers Relat.

[50] Ledermann T, Kenny DA (2017). Analyzing dyadic data with multilevel modeling versus structural equation modeling: a tale of two methods. J Fam Psychol.

[51] Wallhagen MI, Strawbridge WJ, Shema SJ, Kaplan GA (2004). Impact of self-assessed hearing loss on a spouse: a longitudinal analysis of couples. J Gerontol B Psychol Sci Soc Sci.

