# Supplementary material for: Coping with age-related hearing loss: patient-caregiver dyad effects on quality of life
Source: Health Qual Life Outcomes. 2019 May 22;17:86. doi: 10.1186/s12955-019-1161-6 (PMC6532176; doi:10.1186/s12955-019-1161-6)
Supplement: Supplementary file 1 — Dimensions and items of the French four-factor structure of the Brief COPE inventory. (DOCX 30 kb) [file 12955_2019_1161_MOESM1_ESM.docx]

**Supplementary material. Dimensions and items of the French four-factor structure of the Brief COPE inventory**

| I've been getting comfort and understanding from someone | Social support |
| --- | --- |
| I’ve been getting help and advice from other people | Social support |
| I've been saying things to let my unpleasant feelings escape | Social support |
| I've been getting emotional support from others | Social support |
| I’ve been trying to get advice or help from other people about what to do | Social support |
| I've been expressing my negative feelings | Social support |
| I've been praying or meditating | Social support |
| I've been trying to find comfort in my religion or spiritual beliefs | Social support |
| I've been taking action to try to make the situation better | Problem solving |
| I've been concentrating my efforts on doing something about the situation I'm in | Problem solving |
| I've been trying to come up with a strategy about what to do | Problem solving |
| I've been thinking hard about what steps to take | Problem solving |
| I've been using alcohol or other drugs to help me get through it | Avoidance |
| I've been using alcohol or other drugs to make myself feel better | Avoidance |
| I’ve been criticizing myself | Avoidance |
| I’ve been blaming myself for things that happened | Avoidance |
| I've been refusing to believe that it has happened | Avoidance |
| I've been saying to myself "this isn't real" | Avoidance |
| I've been doing something to think about it less0, such as going to movies0, watching TV… | Avoidance |
| I've been giving up the attempt to cope | Avoidance |
| I've been turning to work or other activities to take my mind off things | Avoidance |
| I've been giving up trying to deal with it | Avoidance |
| I've been making jokes about it | Positive thinking |
| I've been making fun of the situation | Positive thinking |
| I've been learning to live with it | Positive thinking |
| I've been accepting the reality of the fact that it has happened | Positive thinking |
| I've been trying to see it in a different light0, to make it seem more positive | Positive thinking |
| I've been looking for something good in what is happening | Positive thinking |
